# Supplementary material for: Effectiveness and Safety of Ayurvedic Medicines in Type 2 Diabetes Mellitus Management: A Systematic Review and Meta-Analysis
Source: Front Pharmacol. 2022 Jun 8;13:821810. doi: 10.3389/fphar.2022.821810 (PMC9213670; doi:10.3389/fphar.2022.821810)
Supplement: Supplementary file 1 [file DataSheet1.zip › Supplementary Material/Forest and Funnel Plots/Zingiber officinale/TG.pdf]

Zingiber officinale - TG

Study	Intervention N	Intervention Mean	Intervention SD	Comparator N	Comparator Mean	Comparator SD	Mean Diff. with 95% CI	Weight (%)
Mahluji 2013	28	127.70	43.70	30	128.20	37.70	-0.50 [-21.46, 20.46]	25.28
Arablou 2014a, Arablou 2014b	33	141.50	47.40	30	190.00	115.30	-48.50 [-91.31, -5.69]	10.03
Azimi 2014, Azimi 2016	41	397.69	51.80	39	382.48	79.44	15.21 [-14.04, 44.46]	17.39
Mozaffari-Khosravi 2014, Talaei 2017, Talaei 2018	40	174.29	82.08	41	172.20	81.81	2.09 [-33.60, 37.78]	13.23
Arzati 2017, Zarezadeh 2018	23	148.00	66.90	22	151.00	70.90	-3.00 [-43.26, 37.26]	11.04
Carvalho 2020	47	174.19	94.44	56	217.71	133.18	-43.52 [-88.94, 1.90]	9.12
Gholinezhad 2020	21	132.66	67.90	21	147.85	43.32	-15.19 [-49.64, 19.26]	13.92
**Overall**							-8.48 [-23.70, 6.73]	

Heterogeneity: $\tau^2 = 124.04$, $I^2 = 30.06\%$, $H^2 = 1.43$

Test of $\theta_i = \theta_j$: Q(6) = 9.18, p = 0.16

Test of $\theta = 0$: z = -1.09, p = 0.27

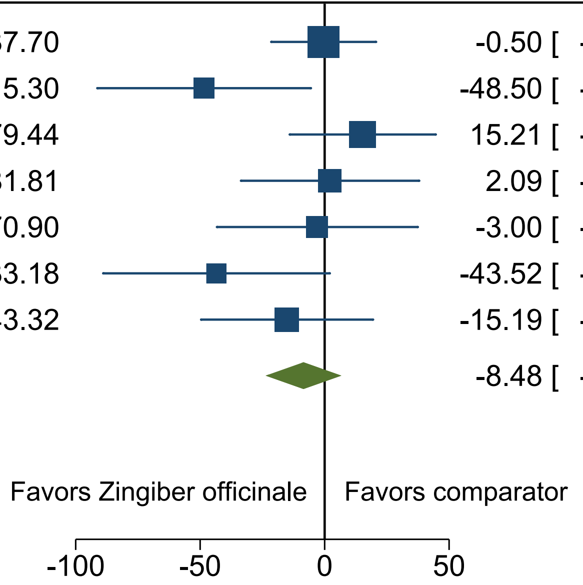

Random-effects REML model
